# Experimental acoustic characterization of an endoskeletal antibubble contrast agent: First results

**DOI:** 10.1002/mp.15242

**Published:** 2021-10-14

**Authors:** Anastasiia Panfilova, Peiran Chen, Ruud J.G. van Sloun, Hessel Wijkstra, Michiel Postema, Albert T. Poortinga, Massimo Mischi

**Affiliations:** ^1^ Electrical Engineering Department Faculty of Electrical Engineering Eindhoven University of Technology Eindhoven The Netherlands; ^2^ Department of Urology Amsterdam University Medical Centers location AMC Amsterdam The Netherlands; ^3^ School of Electrical and Information Engineering University of the Witwatersrand, Johannesburg Braamfontein South Africa; ^4^ BioMediTech Faculty of Medicine and Health Technology Tampere University Tampere Finland; ^5^ Mechanical Engineering Department Eindhoven University of Technology Eindhoven The Netherlands

**Keywords:** harmonic imaging, linear attenuation, nonlinear scattering, pickering‐stabilised bubbles, ultrasound contrast agent

## Abstract

**Purpose:**

An antibubble is an encapsulated gas bubble with an incompressible inclusion inside the gas phase. Current‐generation ultrasound contrast agents are bubble‐based: they contain encapsulated gas bubbles with no inclusions. The objective of this work is to determine the linear and nonlinear responses of an antibubble contrast agent in comparison to two bubble‐based ultrasound contrast agents, that is, reference bubbles and SonoVue${}^{\mathrm{TM}}$.

**Methods:**

Side scatter and attenuation of the three contrast agents were measured, using single‐element ultrasound transducers, operating at 1.0, 2.25, and 3.5 MHz. The scatter measurements were performed at acoustic pressures of 200 and 300 kPa for 1.0 MHz, 300 kPa, and 450 kPa for 2.25 MHz, and 370 and 560 kPa for 3.5 MHz. Attenuation measurements were conducted at pressures of 13, 55, and 50 kPa for 1.0, 2.25, and 3.5 MHz, respectively. In addition, a dynamic contrast‐enhanced ultrasound measurement was performed, imaging the contrast agent flow through a vascular phantom with a commercial diagnostic linear array probe.

**Results:**

Antibubbles generated equivalent or stronger harmonic signal, compared to bubble‐based ultrasound contrast agents. The second harmonic side‐scatter amplitude of the antibubble agent was up to 3 dB greater than that of reference bubble agent and up to 4 dB greater than that of SonoVue${}^{\mathrm{TM}}$ at the estimated concentration of $8\times 10^{4}$ bubbles/mL. For ultrasound with a center transmit frequency of 1.0 MHz, the attenuation coefficient of the antibubble agent was 8.7 dB/cm, whereas the attenuation coefficient of the reference agent was 7.7 and 0.3 dB/cm for SonoVue${}^{\mathrm{TM}}$. At 2.25 MHz, the attenuation coefficients were 9.7, 3.0, and 0.6 dB/cm, respectively. For 3.5 MHz, they were 4.4, 1.8, and 1.0 dB/cm, respectively. A dynamic contrast‐enhanced ultrasound recording showed the nonlinear signal of the antibubble agent to be 31% greater than for reference bubbles and 23% lower than SonoVue${}^{\mathrm{TM}}$ at a high concentration of $2\times 10^{6}$ bubbles/mL.

**Conclusion:**

Endoskeletal antibubbles generate comparable or greater higher harmonics than reference bubbles and SonoVue${}^{\mathrm{TM}}$. As a result, antibubbles with liquid therapeutic agents inside the gas phase have high potential to become a traceable therapeutic agent.

## 1 INTRODUCTION

Ultrasound contrast agents (UCAs) are utilized in the clinic to visualize the blood pool and assess organ perfusion and dispersion, aiding cancer detection.^1^, ^2^, ^3^, ^4^ In some cases, the current UCAs do not exhibit sufficient nonlinear behavior to eliminate clutter and image artifacts, leading to diagnostic misinterpretation.^5^ Augmenting UCA nonlinear behavior improves image contrast and diagnostic confidence. To this end, we propose antibubbles as a new UCA. Endoskeletal antibubbles^6^, ^7^ have been shown to oscillate significantly more asymmetrically than reference bubbles with no cores, and therefore, are hypothesized to demonstrate enhanced nonlinear behavior compared to bubble‐based UCAs.

UCAs are gas microbubbles stabilized by a shell composed of lipids, cross‐linked polymers, or denatured proteins.^8^, ^9^ With a size comparable to that of the red blood cells, they are able to pass through the smallest capillaries. At the same time, they are bigger than endothelial gaps and therefore do not extravasate into tissue.^10^ When insonified at sufficient pressure, gas microbubbles oscillate in a nonlinear fashion, generating higher harmonics.^11^, ^12^ This effect is generally more pronounced when the sonicating frequency is close to the resonance frequency of the microbubbles. The generation of higher harmonics in tissue is much weaker compared to that in UCAs. This enables the implementation of contrast‐specific imaging solutions for visualization of the blood pool, and therefore, analysis of blood flow and vasculature by contrast‐enhanced ultrasound (CE‐US)^13^ and dynamic contrast‐enhanced ultrasound (DCE‐US).^2^, ^4^, ^14^ Despite the recognized utility of CE‐US and DCE‐US in the clinic,^13^, ^15^ cumulative nonlinear effects occurring in tissue can reduce the contrast‐to‐tissue ratio, especially at greater depth.^5^ Several contrast‐specific imaging schemes, such as power modulation^1^, ^16^ and subharmonic imaging,^17^, ^18^ can significantly suppress the nonlinear signal generated by tissue. In this work, we investigate the possibility of using a contrast agent with augmented nonlinear behavior for this purpose, enabling imaging at lower pressure amplitudes and causing weaker higher harmonic generation in tissue.

**FIGURE 1:**
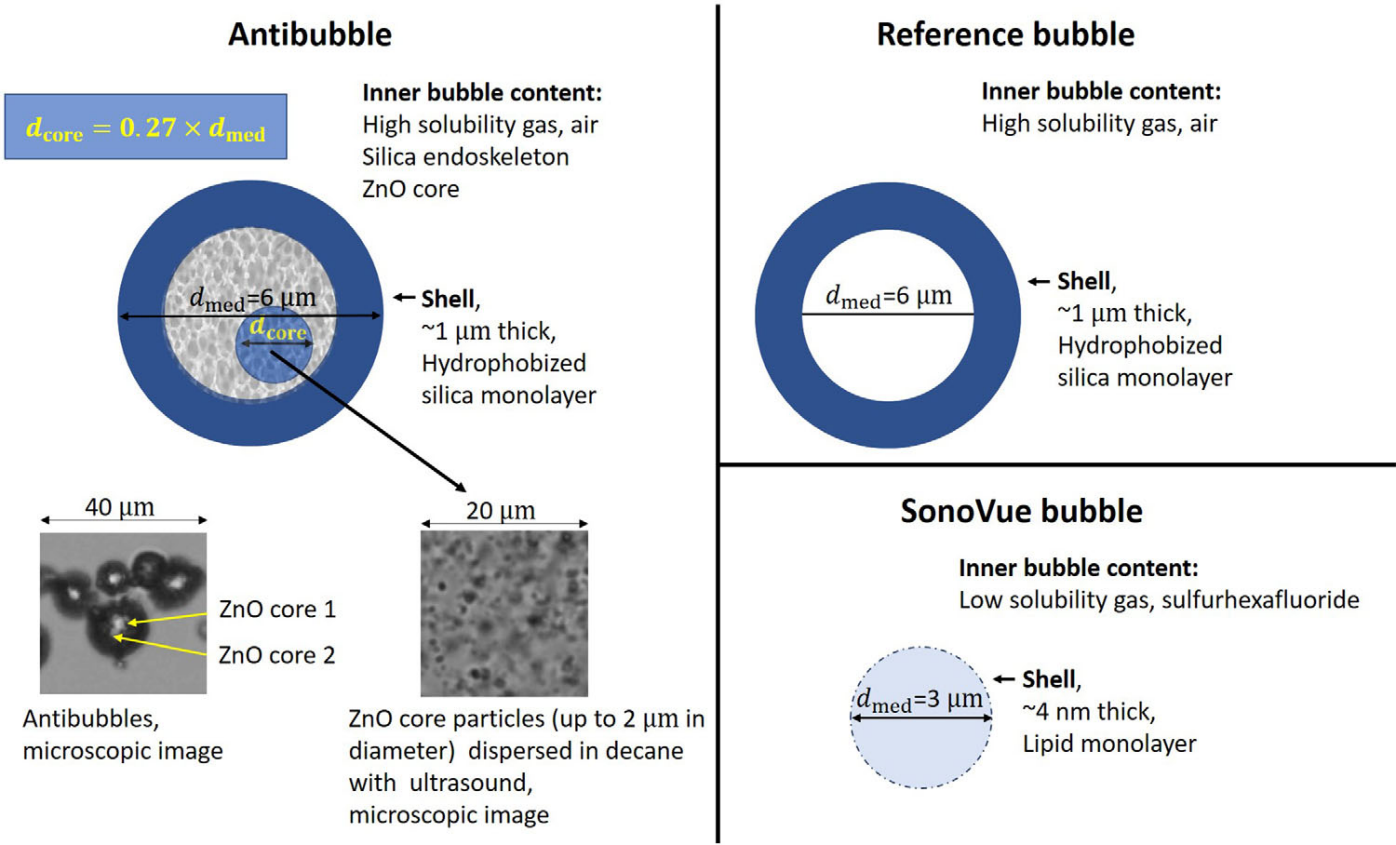
Schematic illustration of antibubbles, reference, and SonoVue${}^{\mathrm{TM}}$ bubbles. The median antibubble diameter was estimated to be 6μm.^24^ The incompressible core(s) comprises, on average, 2% volume, equivalent to an inclusion radius constituting 27% of the bubble radius. For illustration purposes the core particles were dispersed in decane, shown on the inlay. The actual antibubbles do not comprise decane. Surrounding the core is the endoskeleton, encapsulated by a silica shell, whose thickness was estimated to be around 1μm, estimated from microscopic images in Ref. 7. The median reference bubble diameter was estimated to be 6μm.^24^ Reference bubbles are filled with air, encapsulated by a silica shell. SonoVue${}^{\mathrm{TM}}$ bubble, with hexafluoride (${\mathrm{SF}}_{6}$) gas, encapsulated by a thin phospholipid shell^25^, ^26^ and a mean diameter of 3μm^25^, ^27^

A droplet entrapped in a gas bubble has been referred to as an antibubble.^19^, ^20^ This term has also been used for UCAs with microbubbles containing incompressible inclusions in the gas phase.^6^, ^21^ Encapsulated microbubbles that contain incompressible inclusions and a solid supporting skeleton that suspends the inclusion have been referred to as endoskeletal antibubbles.^7^ Figure 1 presents a schematic of an endoskeletal antibubble with a 2% volume inclusion. Theoretical work^21^ demonstrated antibubbles to show an increased nonlinear behavior, compared to reference gas bubbles without incompressible cores. This was attributed to nonsymmetric oscillation in the US field, where the antibubble showed larger expansion than contraction because of the incompressible core. Experimental evidence of this effect was acquired with a high‐speed camera for endoskeletal antibubbles.^7^ These studies^7^, ^21^ suggest that antibubbles have a high potential to improve CE‐US image quality, generating greater higher harmonics, compared to conventional UCAs. Besides this, antibubbles incorporating therapeutic agents in the gas phase can deliver larger doses of therapeutic agents, compared to alternative strategies.^8^, ^22^ This way, antibubbles may offer clinicians a traceable and highly effective therapeutic agent. Currently, no clinically approved antibubble contrast agent exists. Moreover, experimental proof of greater higher harmonic generation is scarce.^23^

**FIGURE 2:**
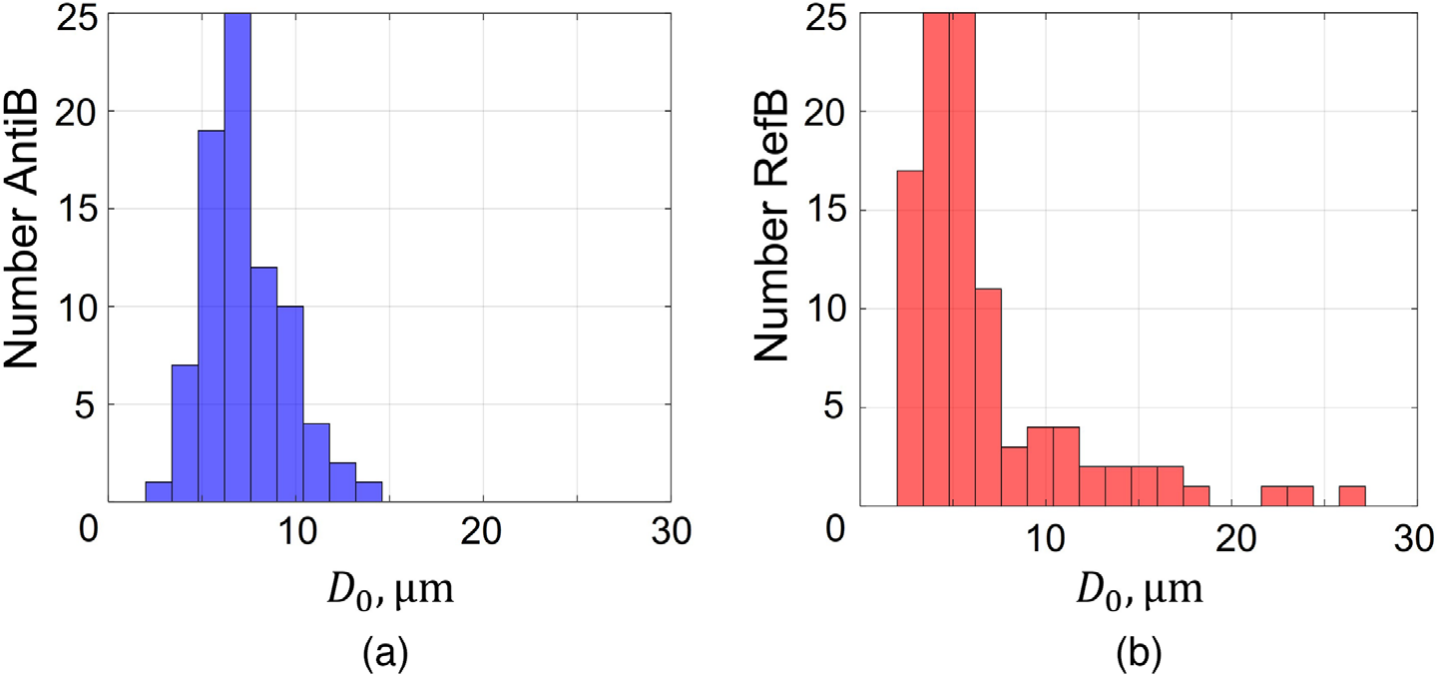
The size distributions of (a) antibubble and (b) reference bubble in the studied dispersions. The data were taken from Anderton^24^

The current experimental study investigated the potential of antibubbles for imaging purposes. To this end, nonlinear side scatter and linear attenuation measurements of endoskeletal antibubbles, reference bubbles, and SonoVue${}^{\mathrm{TM}}$ (Bracco Suisse, Geneva, Switzerland) were performed in the clinically diagnostic frequency range at frequencies of 1.0, 2.25, and 3.5 MHz. The scatter and attenuation parameters quantify UCA efficacy: the amplitude of the nonlinear scatter signal defines the signal‐to‐noise ratio in DCE‐US imaging, whereas attenuation defines the visible image depth.^28^ The endoskeletal antibubbles have a median diameter of 6μm, with 93% smaller than 10μm^24^ (Figure 2a). They are stabilized by a silica shell and contain solid ZnO inclusions in the air gas phase. These inclusions take up 2% of the antibubble volume, whereas the rest of the gas phase contains silica nanoparticles, forming the endoskeleton^7^ (Figure 1). The studied endoskeletal antibubbles are somewhat bigger than those typically utilized in the clinic, and therefore, are currently only a UCA prototype. Reference bubbles have no core inclusions and no endoskeleton but, like antibubbles, have a silica shell^7^ (Figure 1). The median diameter of reference bubbles is 6μm, with 81% below 10μm^24^ (Figure 2b). The endoskeletal antibubbles and reference bubbles have not been clinically approved. Endoskeletal antibubbles are currently only an antibubble prototype, whereas reference bubbles give the opportunity to compare higher harmonic generation of endoskeletal antibubbles to that of a UCA with the same shell. SonoVue${}^{\mathrm{TM}}$ is a clinically approved UCA, used as a benchmark in this study. SonoVue${}^{\mathrm{TM}}$ microbubbles contain a low‐solubility gas (${\mathrm{SF}}_{6}$) encapsulated in a phospholipid shell, with a mean diameter of 3μm, with more than 90% of the bubbles below 8μm^25^, ^27^ (Figure 1). Its size distribution leads to a resonance frequency around 3 MHz.^29^, ^30^ The resonance frequencies of endoskeletal antibubbles and reference bubbles have not yet been identified.

The scatter measurement was performed at acoustic pressures comparable to those employed clinically at mechanical indexes (MIs) of 0.2 and 0.3, often utilized for DCE‐US.^31^, ^32^, ^33^ Signals at these MIs are sufficiently strong to trigger nonlinear bubble oscillation and, at the same time, low enough not to induce damage to biological tissue and bubble bursting.^34^, ^35^ A hydrophone was utilized as a receiver to enable registration of a broad spectrum of the scattered signal, including higher harmonics and subharmonics. The attenuation measurement was performed for MI<0.04. Low pressures are typical for attenuation measurements,^36^ and they avoid depletion of the fundamental signal due to generation of higher harmonics, which is especially prominent in case of UCAs. Moreover, at greater pressures, UCA bubbles may generate a strong fundamental component, interfering with the transmitted pulse and introducing further error in the measurement. This way, we measured attenuation as a result of energy absorption^28^ and energy scattering in a nearly linear low‐amplitude regime of bubble oscillation,^37^ as demonstrated for antibubbles, reference bubbles, and other contrast agents at greater pressures than those utilized in this work.^7^, ^38^, ^39^ For both scatter and attenuation measurement, pulses of 10–20 cycles were transmitted, providing a sufficiently narrow bandwidth to avoid overlap between the harmonics in the received spectra.

To demonstrate antibubbles' performance in a nearly clinical setting, a DCE‐US measurement was performed: the flow of endoskeletal antibubbles, reference bubbles, and SonoVue${}^{\mathrm{TM}}$ was imaged through a porous phantom^40^ with a linear array US transducer. The spaces between the acoustically transparent beads composing the vascular phantom simulated a vascular network. The transmitted pulses consisted of three cycles at 3.5 MHz, granting sufficient resolution for phantom visualization, and with an MI = 0.2.

## 2 METHODS

### 2.1 Scatter measurements

#### 2.1.1 Contrast agent fabrication and preparation

Endoskeletal antibubbles were produced as described by Poortinga^6^ with some modifications. The antibubbles were stabilized using pharmaceutical grade Aerosil^®^ 972 Pharma hydrophobized silica particles (Evonik Industries AG, Essen, Germany). The aqueous cores were replaced by 2 vol% (sample 1) of hydrophobically modified Zano 10 Plus ZnO nanoparticles (Umicore, Brussels, Belgium). Reference bubbles containing no cores were produced in the same way but without adding core material. As compared to the procedure in Ref. 6, the mixing speed of the high‐shear mixer (IKA T18 Ultra Turrax equipped with an S18N‐19G rotor stator) was increased to 12 000 rotations per minute to produce smaller antibubbles with a size comparable to that of conventional UCAs.

All UCAs were maintained at room temperature before activation. The concentrations of investigated UCA dispersions were chosen in the low range, where a linear dependence of the scattered energy^28^, ^39^, ^41^, ^42^ and attenuation^28^, ^43^ on bubble concentration has been reported. The adopted concentration was in the order of $10^{5}$ bubbles/mL for all studied UCAs, yielding a sufficient signal‐to‐noise ratio of the received signals. Preparation of reference bubble and antibubble dispersions was identical. Ten milligram of dried material was diluted with 12 mL of saline in a vial. The resulting dispersions was manually gently agitated for 10 s, and 0.35 mL was taken out and diluted in 3.35 mL of saline, giving a concentration of 0.08 mg per 1 mL. This corresponded to $10^{5}$ bubbles/mL, based on the calculation of the overall gas volume corresponding to 0.08 mg of powder material and the average bubble volume. SonoVue${}^{\mathrm{TM}}$ was prepared according to the manufacturer instructions. The vial was gently agitated, and 0.4 mL of the dispersions was extracted right before the measurement and diluted to the concentration of 1μg per 1 mL that corresponds to $8\times 10^{4}$ bubbles/mL. For all UCAs, the final dispersions was gently shaken manually for 10 s right before the measurement.

#### 2.1.2 Experimental procedure

**FIGURE 3:**
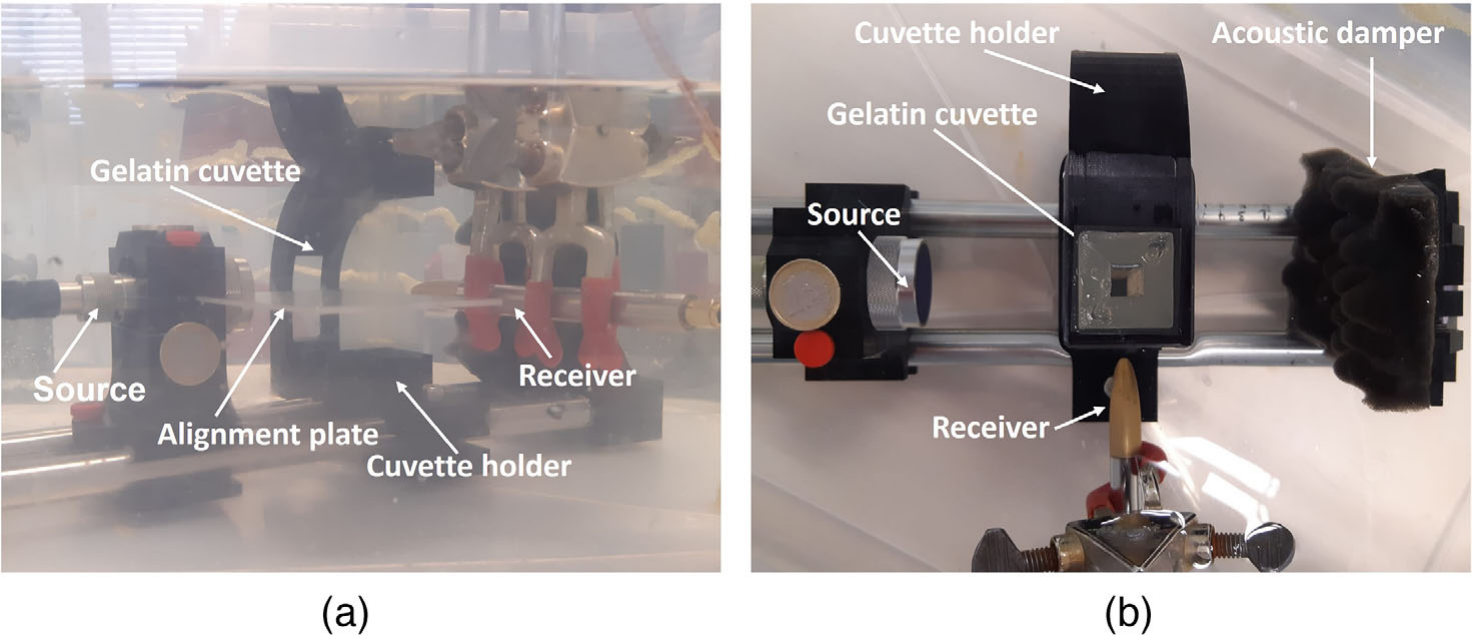
The setup for scatter measurements: (a) side view and (b) top view

The center of the cuvette with UCA dispersions was always positioned a few millimeters beyond the focal point of an US source (Figure 3). A source holder and a cuvette holder were fixed on a rail system, providing alignment of the source and the cuvette. The utilized sources were all single‐element focused US circular transducers with a diameter of 2.5 cm, and a focal distance of 6.4±0.1 cm. Transducers of type V302‐SU‐F, V304‐SU‐F, and V380‐SU‐F (Olympus Nederland B.V., Leiderdorp, the Netherlands), were excited at their center frequencies of 1.0, 2.25, and 3.5 MHz, respectively. The omnidirectional secondary field created by the UCAs was recorded with a HGL‐0400 S/N 1037 1.0 mm capsule hydrophone (Onda Corp., Sunnyvale, CA, USA), oriented perpendicular to the source (Figure 3). This is a typical configuration of the source and the receiver, utilized for scatter measurements.^12^, ^38^, ^42^, ^44^

Gelatin cuvettes were prepared to contain the contrast agents during the experiments. Their advantage is a similar acoustic impedance to that of water, and therefore, low reflection. For cuvette fabrication, a hollow form and a ceiling top were 3D printed. The black cubic form was hollow, with inner dimensions of 12 × 3 × 3 cm^3^. A lid was printed to seal the top of the cuvette once the liquid gelatin mixture was inside. This lid had a long cubic stick in the center with dimensions 10 × 1 × 1 cm^3^. This construction shaped cubic gelatin cuvettes with 1 cm thick sides, and a hollow cubic void (with the dimensions of the cubic stick), where contrast material could be poured in. When preparing the gelatin mixture, eight sheets of animal‐based gelatin (Dr. Oetker, Amersfoort, The Netherlands) were diluted in 100 mL of water, corresponding to a concentration of 13 g of gelatin per 100 mL. The mixture was poured in the hollow form, smeared with baseline from the inside. The form was sealed with the lid and put in the fridge.

Prior to the scatter measurements, the voltage amplitudes of the driving signals were identified for each transducer to generate MIs of 0.2 and 0.3. These MIs are often used in clinical practice because they do not induce either bubble bursting or damage to biological tissue.^34^, ^35^ Nevertheless, such signals are sufficiently strong to trigger nonlinear bubble oscillation. In the MI measurement, the gelatin cuvette was modified to position the hydrophone in the center of the cuvette. The generated pressures were recorded for various voltage amplitudes of the signals driving the source. The initial placement of the hydrophone in the center of the cuvette was visually aided, and further adjustments were performed with the help of an oscilloscope, identifying the angular orientation of the hydrophone with the maximum signal amplitude.

Before the scatter measurement, the setup was submerged in a degassed water bath and left for a half an hour, allowing the gelatin phantom extracted from the fridge to reach room temperature. Two sides of the water tank, facing the source, were lined with foam to reduce possible reflections. The first measurement was always conducted for the reference liquid of saline. Additional acquisitions with a needle on the inside borders of the cuvette were acquired to identify the region where the UCA signal was expected to originate. Further, the cuvette was emptied, and the contrast‐agent dispersions were gently shaken and slowly injected in the cuvette. The UCA was shaken to ensure a homogeneous dispersion, that is, with a homogeneous spatial distribution of bubbles/antibubbles, filling the whole inner cavity of the cuvette. The measurement was performed right after injection not to allow the larger bubbles with more gas to rise. Two driving voltages were used one after another, corresponding to MI = 0.2 and MI = 0.3, in an ascending order. The whole measurement lasted for a few seconds. This procedure was repeated 15 times for each UCA, with different batches of the contrast‐agent dispersions being injected in the cuvette.

Labview (National Instruments Corp., Austin, TX, USA) was used to control the US acquisition of the acoustic response generated by UCAs. A 33220A arbitrary wave generator (Agilent Technologies, Santa Clara, California, USA) was connected to a desktop and controlled by dedicated Labview software to generate the driving signals. The driving signals were transmitted to a 50‐dB 2100L RF Power amplifier (Acquitek, Massy, France) connected to the source transducer. The received signals were displayed on a TDS2014 oscilloscope (Tektronix U.K. Limited, Bracknell, UK) and were further sampled throughout an NI‐5122 (National Instruments Corp.) acquisition board which was connected back to the desktop and controlled by the Labview software. Sinusoidal tone bursts with a rectangular window, were transmitted by 3 sources driven at their center frequencies of 1.0, 2.25, and 3.5 MHz. A length of 20 cycles was chosen for 2.25 MHz and 3.5 MHz, and 10 cycles for 1.0 MHz. These pulse lengths provided a sufficiently narrow bandwidth of the transmitted signals and, therefore, allowed avoiding overlap between the harmonics in the received spectrum. The silence period between the pulses was always set to 250 μs, chosen to prevent interference of any possible reflection of the preceding pulse with the following pulse. A total of 92–95 pulses were transmitted at every acquisition. The signals were recorded at a sampling frequency of 25 MHz and stored for analysis.

#### 2.1.3 Data analysis of scatter measurements

All data analysis was performed with MATLAB^®^(The MathWorks, Inc., Natick, MA, USA). In the scatter measurement, the analyzed segment of the signal, generated by the UCAs, was identified manually, confirmed by the acquisitions with the needles inside the cuvette (Section 2.1.2). A window of  9 μs was chosen for the analysis, defined by the shortest signal generated by the UCAs. The length of the time window was fixed for all sonicating frequencies. For each acquisition, an average Fourier amplitude spectrum was calculated based on 92 acquisitions, radiated by the UCAs, using Matlab's Fast Fourier transform function. A Hanning window was used in all cases to reduce spectral leakage.^45^ The nonlinear scatter spectra were represented in two ways. First, using

(1) $$S_{\mathrm{nonl}}\left(f\right)=10\log_{10}\left(\frac{A_{\mathrm{CA}}\left(f\right)}{A_{\mathrm{sal}}\left(f\right)}\right),$$

where ${A(f)}_{\mathrm{CA}}$ is the amplitude of the signal generated by the UCA at frequency f and ${A(f)}_{\mathrm{sal}}$ is the amplitude of the signal at frequency f acquired with saline in the cuvette, representing the noise level at that frequency.^23^ In the second representation, the spectra were normalized by the amplitude of the fundamental frequency $A_{\mathrm{CA}}\left(f_{0}\right)$ in these spectra, using

(2) $$S_{\mathrm{nonl}}\left(f\right)=10\log_{10}\left(\frac{A_{\mathrm{CA}}\left(f\right)}{A_{\mathrm{CA}}\left(f_{0}\right)}\right).$$

For each medium studied, the responses of 15 acquisitions, calculated with Equations (1) and (2), were averaged, and their standard deviation was assessed.

Equation (1) allows comparison of the UCA efficacy at the concentrations used. However, this concentration is significantly lower than that used in the clinic.^46^, ^47^ As in the low concentration range, all the generated harmonic amplitudes are proportional to the bubble concentration,^39^, ^42^ we normalized the UCA responses at all frequencies by the corresponding responses at the fundamental frequency, as shown in de Jong et al.^30^ Besides this, the scattered energy for every harmonic is also proportional to the squared bubble radius.^42^ Therefore, it is hypothesized that this normalization reduces the influence of bubbles size and concentration on the scattered spectra.

Assessment of the significance of the differences between the higher harmonics of the UCAs was performed with the two‐tailed Student's *t*‐test, assuming a Gaussian distribution of the higher harmonic amplitudes among the 15 acquisitions. A *p*‐value below 0.05 is considered to indicate a statistically significant difference between the distributions.

### 2.2 Attenuation measurements

**FIGURE 4:**
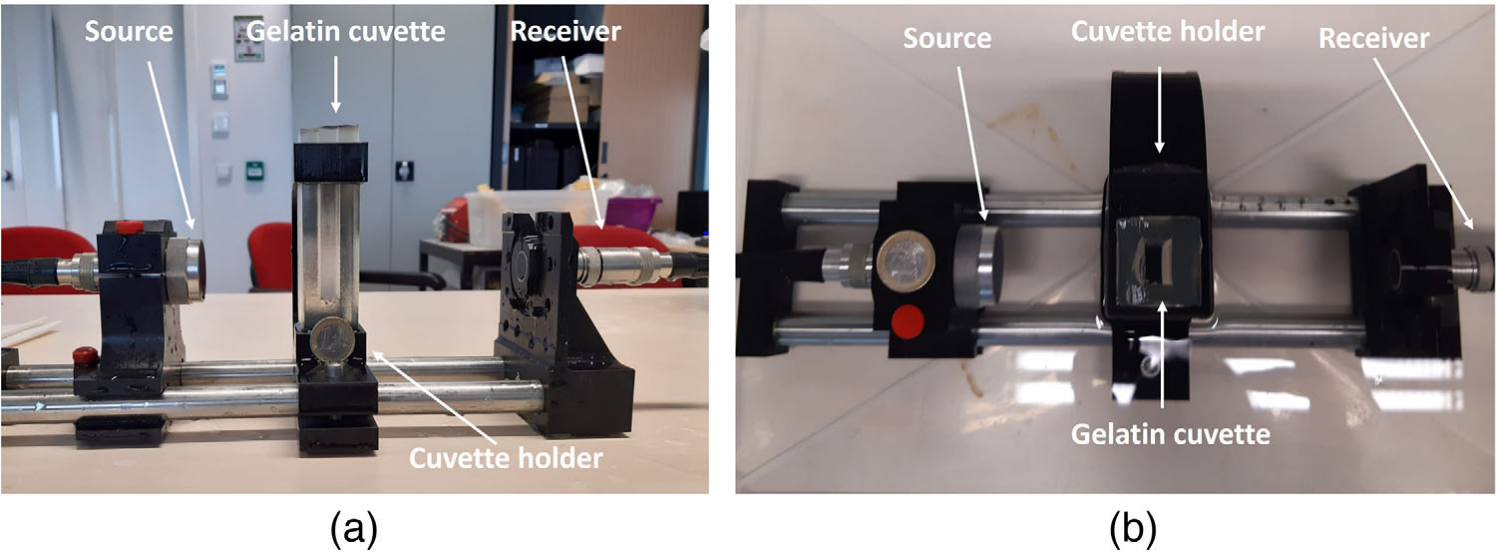
The setup for attenuation measurements: (a) side view of the setup taken out of the water bath and (b) top view

The attenuation measurement was performed with a transmission setting (Figure 4). The same source transducers as for the scatter measurement (Section 2.1) were employed, resulting in attenuation coefficients at three frequencies for all studied UCAs. The sources, the receivers, and the cuvette with UCAs were fixed on the same rail system as for the scatter measurement. The center of the cuvette was positioned in the focal region of the sources. The opening in the gelatin cuvette containing UCA was 1.6‐cm wide, ensuring that the whole beam passed through the studied dispersions. The length of the cuvette, corresponding to the path in the UCA dispersion, was 1 cm. Varying greatly among other experimental studies, from 2 to 8 cm,^38^, ^48^, ^49^, ^50^ this cuvette length was chosen to ensure a sufficient signal‐to‐noise ratio after propagation through all studied UCAs at all frequencies. The receiver was fixed in a holder located 85 mm away from the cuvette (Figure 4). A plane piston V306 transducer (Panametrics‐NDT, Olympus NDT Inc., Waltham, MA, USA), centered at 2.25 MHz, was used as the receiver for measurements at 1.0 and 2.25 MHz, and a plane piston V309 (Panametrics‐NDT, Olympus NDT Inc., Waltham, MA, USA) was used for 3.5 MHz. The same system and control software was used for the attenuation measurement (Section 2.1.2), transmitting 20‐cycle tone bursts for all frequencies. For every source, transmitted pressure amplitudes were below 60 kPa, measured in the center of the cuvette by the hydrophone, as for the scatter measurement (Section 2.1.2). At such low pressures, the bubble oscillation is mostly linear, as demonstrated in (Kudo et al., 2020)^7^ for antibubbles and reference bubbles. SonoVue${}^{\mathrm{TM}}$, on the other hand, exhibited an initiation of second harmonic growth at pressures of 24–50 kPa,^30^, ^51^ depending on the transmit frequency. Therefore, a preliminary experiment was conducted, assessing the higher harmonic amplitudes for the chosen settings, demonstrating the higher harmonics to be below 5% of the fundamental signal. This way, it was concluded that the chosen settings primarily corresponded to the linear regime of bubble oscillation. Contrast‐agent dispersions were prepared as for the scattering measurement (Section 2.1.1). They were injected in a gelatin cuvette right before the measurement. Attenuation was estimated based on two measurements: when the cuvette contained 7 mL of saline and 7 mL of UCA. The Fast Fourier transform was performed on all the received pulses in the acquisitions and the average amplitude at the fundamental frequency was extracted for saline $A_{\mathrm{sal}}$ and UCA $A_{\mathrm{CA}}$. The attenuation coefficient was computed using

(3) $$\alpha \left(f\right)=\frac{20}{d}\log_{10}\left(\frac{TA_{\mathrm{sal}}\left(f\right)}{A_{\mathrm{CA}}\left(f\right)}\right),$$

where d is the length of the US path in the UCA medium defined by the inner dimensions of the cuvette and T is the transmit coefficient.^38^, ^49^ For every UCA, 15 acquisitions were performed, yielding 15 values of the attenuation coefficient. The mean and standard deviation among these acquisitions were assessed.

In our measurement in Equation (3), d=1 cm and T was approximated to 1, neglecting all reflection losses and possible acoustic impedance differences of UCAs with respect to water. In an experiment designed to verify this assumption for our gelatin cuvettes, it was identified that the amplitude of the transmitted pulse decreased by 1% only per 1 cm of the path in gelatin. These losses include reflection loss (defined by the difference in acoustic impedance) and attenuation. As for UCAs, the density was assumed identical to that of water due to the low concentration of bubbles used. The arrival times of the pulses transmitted through UCAs were compared to that in water. A maximum delay among all UCAs corresponded to 0.6μs, indicating a maximum difference of 8% in the speed of sound compared to water. For the three investigated frequencies, an attenuation measurement was conducted for corn oil, utilizing the same setup and making the same assumptions. The attenuation coefficients were in agreement with literature values,^52^ within a ± 0.1 dB/cm error.

To verify the measured attenuation values, another attenuation measurement was conducted with a different set up for 3.5 MHz, at the same acoustic pressure amplitude as for the through‐transmission measurement, corresponding to MI = 0.03. Twenty‐cycle pulses were transmitted, with a Gaussian window. An L11‐4v linear array probe, controlled by a Verasonics US system (Vantage 128, Verasonics Inc., Kirkland, WA, USA), was utilized to construct echo‐mode videos, consisting of 100 frames. The image gain was set constant throughout depth and no log compression was performed; therefore, the gray levels of the videos represented the envelopes of the signals. The probe was positioned to provide normal incidence of the sonicating beam on the cuvette, while an aluminum plate behind the cuvette served as the reflector, as in Ref. 53. A reference measurement with saline in the cuvette was performed, where $A_{\mathrm{sal}}$ was the grayvalue of the aluminum plate (Equation (3)), averaged over the 100 frames. Then the saline was taken out with a syringe and the UCA suspension was injected in the cuvette, resulting in a mean gray level of the aluminum plate $A_{\mathrm{CA}}$. The difference in the grayscale intensity of the aluminum plate in these two measurements allows to compute the attenuation coefficient (Equation (3)), accounting for the longer path through the UCA dispersion due to forward and backward directions. For all UCAs, the attenuation coefficient of six or seven analogous dispersions was measured.

Besides this, the ratio of the pressures backscattered by the UCA suspensions to the transmitted pressure amplitudes was estimated for 3.5 MHz. For this estimation, the cuvette was removed from the acoustic path. The gray level intensity of the aluminum plate divided by the reflection coefficient of the water–aluminum interface represented the sonicating amplitude $A_{\mathrm{transmit}}$. The mean gray level inside the cuvette represented the backscattered energy $A_{\mathrm{backsc}}$. The ratio was computed as

(4) $$S_{\mathrm{lin}}\left(f\right)=\frac{A_{\mathrm{backsc}}\left(f\right)}{A_{\mathrm{transmit}}\left(f\right)}\times 100\%.$$

$S_{\mathrm{lin}}$ represents the linear scatter at the fundamental frequency, because higher harmonic generation at these pressures was demonstrated to be negligible for antibubbles, reference bubbles, and SonoVue${}^{\mathrm{TM}}$.^7^, ^42^, ^44^

### 2.3 Dynamic contrast‐enhanced ultrasound measurement

**FIGURE 5:**
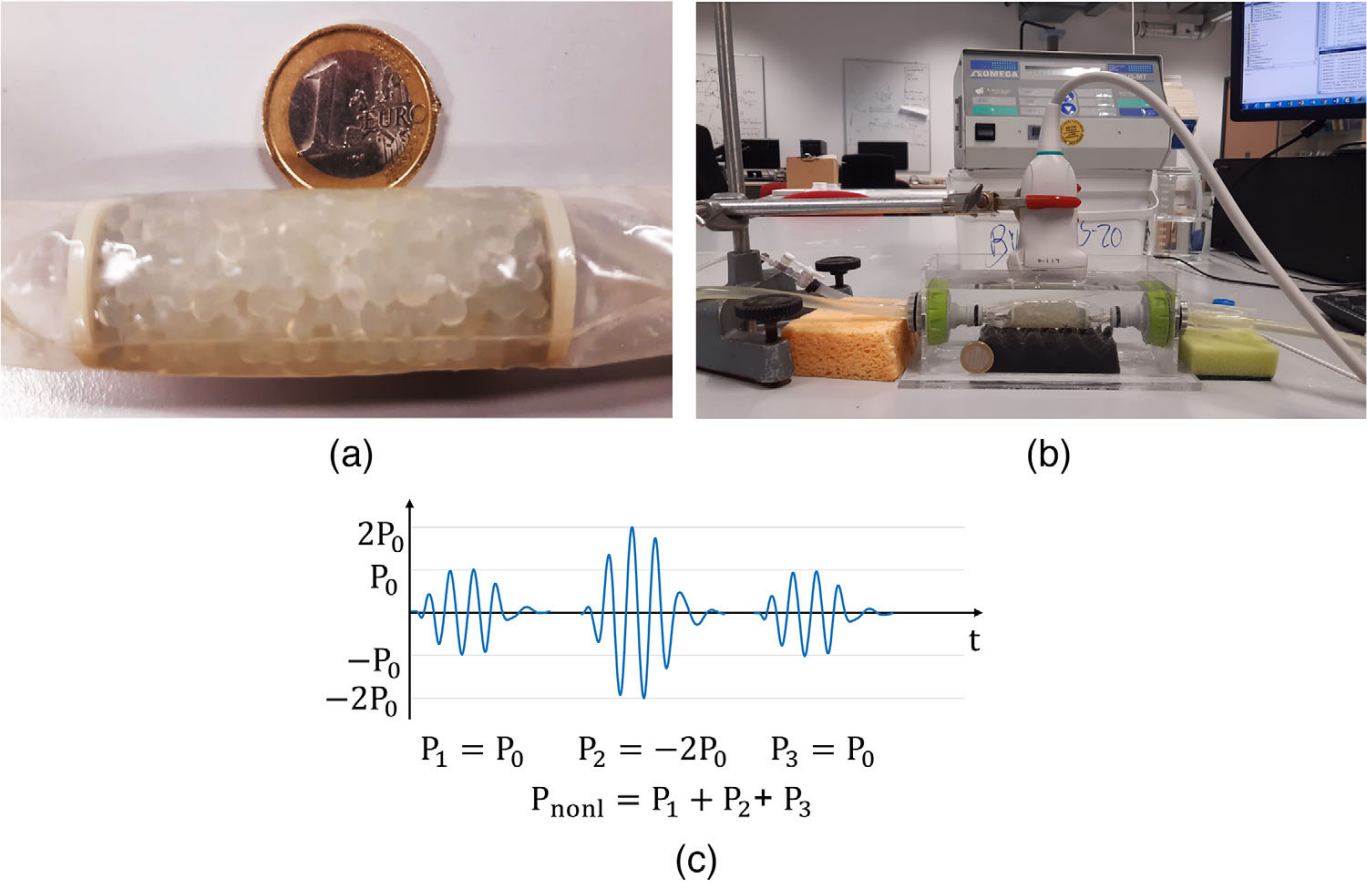
The setup for the dynamic contrast‐enhanced ultrasound measurement. (a) A close‐up view of a vascular phantom made of beads. (b) The probe is mounted on top of the vascular phantom in a water bath. (c) The utilized pulse scheme, where three pulses were transmitted ($P_{1}$, $P_{2}$, and $P_{3}$) to form a DCE‐US clip, reflecting the intensity of the nonlinear signal $P_{\mathrm{nonl}}$

To investigate the efficacy of UCAs in a near to clinical setting, a DCE‐US measurement was performed. In this measurement, the UCAs were separately injected into a perfusion system, flowing through a porous phantom that mimicked a microvascular network. Degassed water flow through the phantom was supplied with an FPU5‐MT peristaltic pump (Omega Engineering Ltd., Manchester, UK) at a rate of 36 mL/min. The utilized flow was in the range of expected physiological values, ranging from 10${}^{-7}$ mL/min for capillaries^54^ to 10${}^{3}$ mL/min for large arteries.^54^, ^55^ The porous phantom (Figure 5a) was built by packing alginate beads with a diameter of 3.1 mm in a polyurethane tube, whose shape was fixed by two circular nets at the two sides of the phantoms. The phantom was gently squeezed and shaken after packing to achieve a more homogeneous structure.^40^ The spaces between the beads simulated a microvascular network with porosity of 43%. As the phantom consisted of identical beads, the simulated microvascular network did not exhibit the vessel/capillary topology typical for biological tissue.^54^, ^56^ The water resistant alginate beads did not permit simulating tissue–water exchange observed in biological tissue.^57^ This way, the phantom provided a simplified model of a microvascular network. The phantom used in the experiments was about 4.5 cm long, comparable to the length of the utilized US transducer, and 2 cm in diameter. Before the experiment, the phantom was submerged in a water bath and connected to the input and output flow paths (Figure 5b). A linear array L11‐4v probe, controlled by a Verasonics US system, was mounted above the phantom. DCE‐US plane wave imaging was performed in contrast‐specific mode following the manual injection of a UCA bolus into the flow stream toward the vascular phantom. The utilized pulse scheme is illustrated in Figure 5(c), consisting of one high‐amplitude pulse and two pulses, twice as low in amplitude and shifted in phase by 180${}^{\circ }$. The high‐amplitude pulse pressure was 370 kPa, corresponding to MI = 0.2 (the probe was calibrated with the same hydrophone). The transmitted pulses consisted of three cycles at 3.5 MHz, granting sufficient resolution for visualization of the beads composing the phantom. The contrast‐specific mode was a combination of pulse inversion and amplitude modulation schemes, because it was shown to be the most sensitive imaging strategy to microbubble nonlinearities.^58^

When injecting the contrast‐agent bolus, we aimed to have the same concentrations at the peak of the time–intensity curves (TICs) as in the static measurement. A few preliminary measurements were conducted where 1 mL of reference bubble and antibubble dispersions (0.5 mg/mL) was injected. The water volume where the UCA was diluted^2^, ^3^ before its arrival to the middle of the vascular phantom was assessed with the measured TICs in the middle of the phantom. A simple triangular model^59^, ^60^ was used to correlate the concentration at the peak of the TIC and the identified mixing volume. For SonoVue${}^{\mathrm{TM}}$, two greater concentrations were also used. As the SonoVue${}^{\mathrm{TM}}$ bubbles are smaller, greater number densities were used to reach a volume fraction comparable to that in the reference and antibubble boluses. In these cases, the concentration of SonoVue${}^{\mathrm{TM}}$ at the peak of the TIC was estimated to be 10 and 30 times greater than the concentration used in the static measurement.

For every UCA, four DCE‐US acquisitions were performed. For every acquisition, a 1‐mL bolus with the calculated concentrations was injected. Forty‐second dynamic contrast‐enhanced US clips recorded the flow through the porous phantom including the complete wash‐in and wash‐out. The TICs were extracted from the middle of the phantom (Figure 9) and compared, with the aim to identify the UCA producing the highest peak signal.

**FIGURE 6:**
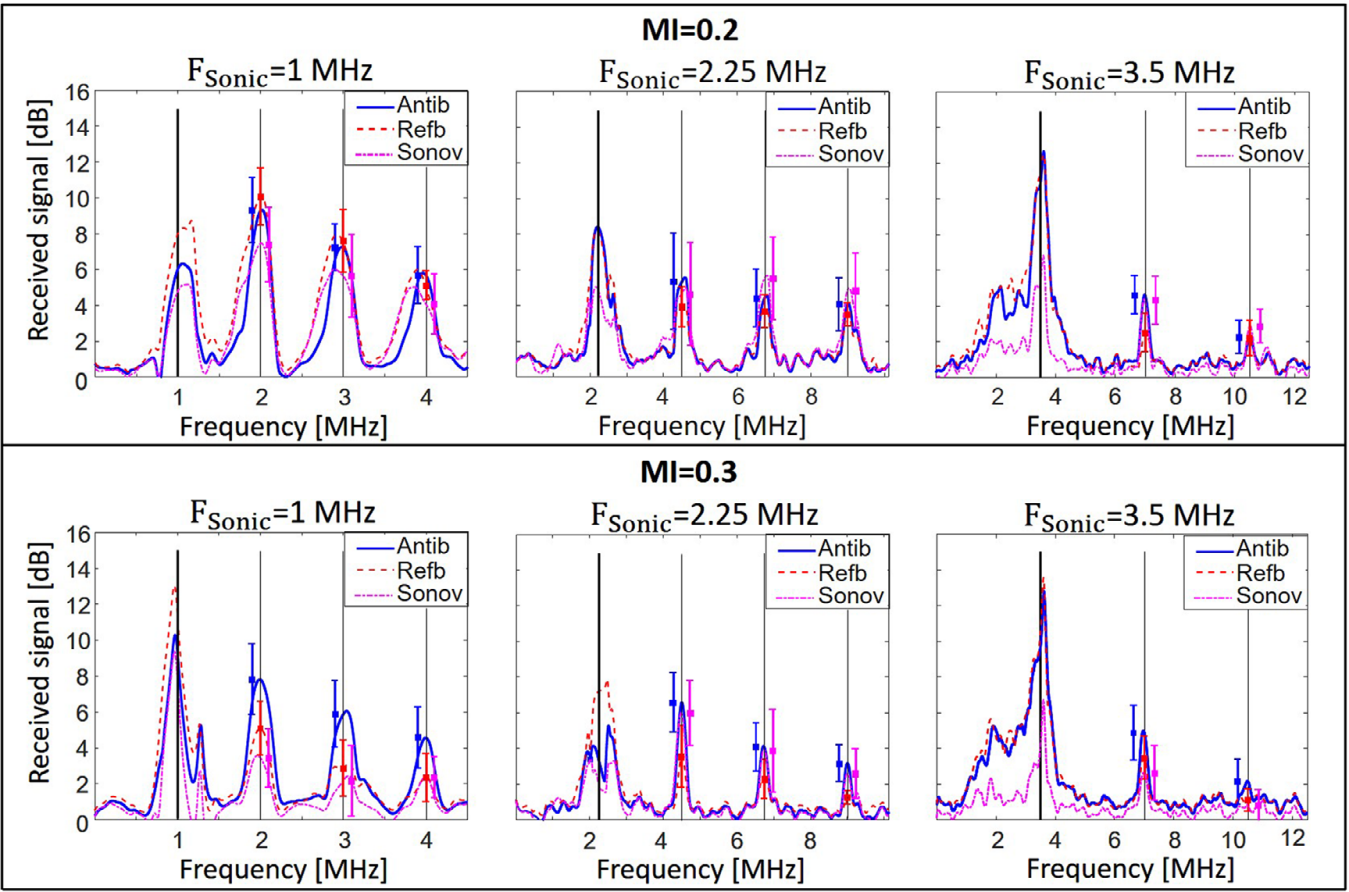
Mean spectra of the signals generated by antibubbles (Antib), reference bubbles (Refb), and SonoVue${}^{\mathrm{TM}}$ (Sonov) at frequencies of 1.0, 2.25, and 3.5 MHz for MIs of 0.2 (top) and 0.3 (bottom). The amplitude of the responses is presented in dB, normalized with respect to the responses in a reference acquisition with saline in the cuvette. The thick vertical lines indicate the fundamental frequency, whereas the thin vertical lines indicate higher harmonics. The error bars represent the standard deviations of the higher harmonic amplitudes

**FIGURE 7:**
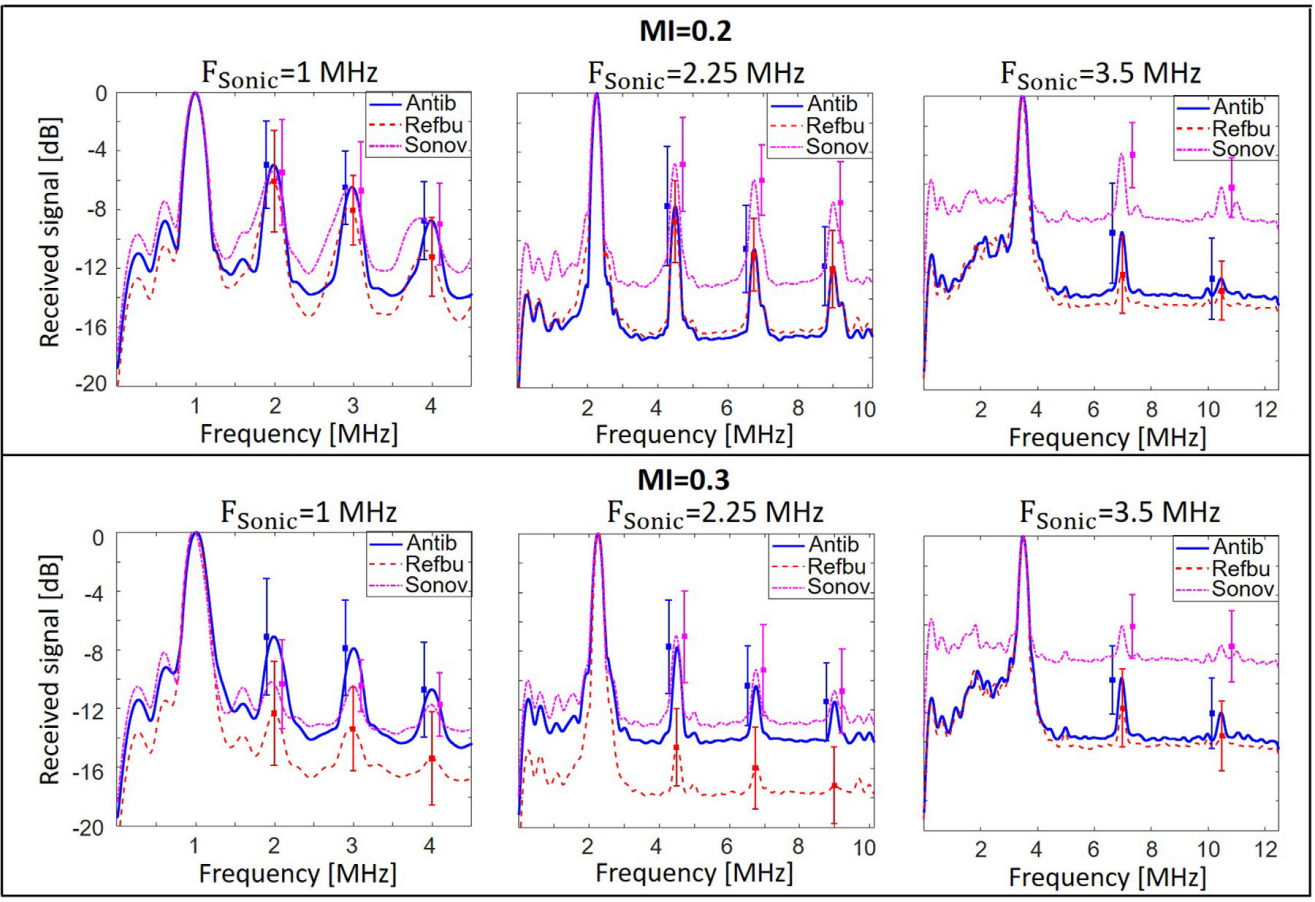
Spectra of the signals generated by UCAs at frequencies of 1.0, 2.25, and 3.5 MHz for MIs of 0.2 and 0.3. The amplitude of the responses is presented in dB, normalized with respect to the fundamental component in these responses

**FIGURE 8:**
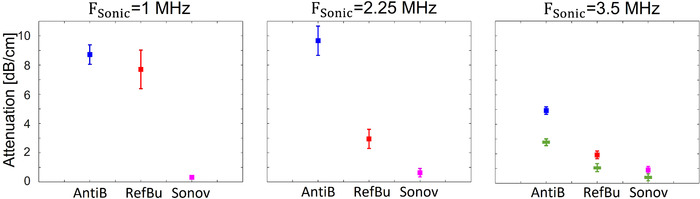
Attenuation coefficients measured for antibubbles (Antib), reference bubbles (Refb), and SonoVue${}^{\mathrm{TM}}$ (Sonov) at sonicating frequencies of 1.0, 2.25, and 3.5 MHz (left to right). The square symbols indicate the mean attenuation coefficients measured with the piston source and receiver (Figure 4). The rectangular (green) symbols indicate the mean attenuation measured in echo mode with the Verasonics probe for 3.5 MHz

**FIGURE 9:**
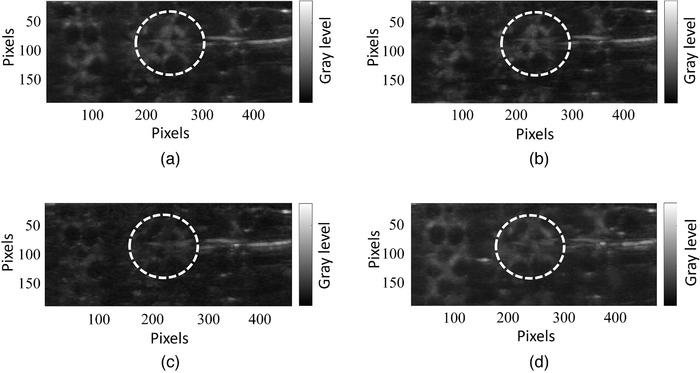
Maximum intensity projection based on the DCE‐US recordings of UCAs passing through the porous phantom. The white contour indicates the region of interest where the TICs were extracted. (a) Antibubbles. (b) Reference bubbles. (c) SonoVue${}^{\mathrm{TM}}$ with a peak concentration as in the static measurement. (d) SonoVue${}^{\mathrm{TM}}$ with a peak concentration 30 times higher than in the static measurement

## 3 RESULTS

### 3.1 Scattering

The average spectra of the signals generated by UCAs, normalized to the saline spectrum, are presented in Figure 6. The frequencies of the sonicating signals are stated above the plots. The thick vertical straight lines on the plots indicate this sonicating frequency, and therefore, the fundamental component of the signal scattered by UCAs. The thinner lines indicate integers of the fundamental frequency, representing the scattered higher harmonics. The error bars next to these lines demonstrate the mean and the standard deviation of the higher harmonic amplitudes among the 15 acquisitions for antibubbles (to the left of the harmonic line, in blue), reference bubbles (on the harmonic line, in red), and SonoVue${}^{\mathrm{TM}}$ (to the right of the harmonic line, in magenta). For the top plots, demonstrating the UCA response to sonication at an MI of 0.2, for 1.0 and 2.25 MHz, the nonlinear responses of antibubbles and reference bubbles are equivalent: the mean higher harmonic amplitudes differ no more than by 2 dB, with no significant differences in the higher harmonic distributions (*p* > 0.05). For 3.5 MHz, the antibubbles scatter a second harmonic 2 dB greater than the reference bubbles, with a significant (*p* < 0.001) difference in the distributions. The third harmonic content is equivalent. For SonoVue${}^{\mathrm{TM}}$, at 1.0 MHz, all higher harmonics are up to 2 dB weaker than those of antibubbles with a significant difference in the higher harmonic distributions (*p* < 0.05). For 2.25 and 3.5 MHz, SonoVue${}^{\mathrm{TM}}$ exhibits an analogous nonlinear response to antibubbles, with barely a difference in higher harmonic amplitudes and complete or great overlap of the confidence intervals (*p* > 0.05). For an MI of 0.3, all higher harmonics generated by antibubbles are significantly (*p* < 0.01) greater than for the reference bubbles at all the sonicating frequencies. In comparison to SonoVue${}^{\mathrm{TM}}$, at 1.0 MHz, the higher harmonic amplitudes of antibubbles are 2–4 dB greater (*p* < 0.001). At 2.25 MHz, the nonlinear responses are equivalent (*p* > 0.05), whereas for 3.5 MHz, the second and third harmonic of antibubbles are significantly (*p* < 0.001) greater (up to 2 dB) than those of SonoVue${}^{\mathrm{TM}}$.

The spectra normalized to their corresponding fundamental signals are shown in Figure 7. Theoretical analysis predicts that all the harmonic amplitudes scattered by a UCA dispersion are proportional to the bubble concentration, in the low concentration range, and the bubble radius.^11^, ^39^, ^42^ Normalizing the spectrum to the fundamental harmonic amplitude is hypothesized to mitigate to some extent the impact of the different bubble sizes and concentrations. This way, the provided normalized plots facilitate comparing the nonlinear behavior of the investigated bubbles/antibubbles.

Analysis of the scatter spectra at pressures corresponding to an MI = 0.2 follows below. At 1.0 MHz, antibubbles generated mean higher harmonic amplitudes equivalent to those of SonoVue${}^{\mathrm{TM}}$, with nearly complete overlap of the confidence intervals (*p* > 0.05). The mean higher harmonic amplitudes of reference bubbles are somewhat lower than those of antibubbles, with the greatest significant (*p* < 0.01) difference of 3 dB in the fourth harmonic. At 2.25 MHz, higher harmonics of antibubbles are up to 5 dB weaker (third harmonic) than those of SonoVue${}^{\mathrm{TM}}$, with a significant difference between the harmonic distributions (*p* < 0.05), and equivalent to reference bubbles (*p* > 0.05), with the antibubble signal up to 1 dB greater than that of reference bubbles. At 3.5 MHz, antibubble higher harmonics are significantly (*p* < 0.001) up to 6 dB weaker than those of SonoVue${}^{\mathrm{TM}}$. At the same time, they are up to 3 dB greater than those of reference bubbles with a significant difference between the scattered higher harmonics (*p* < 0.01).

Analysis of the scatter responses at pressures corresponding to an MI = 0.3 follows below. For 1.0 MHz, antibubble higher harmonics are up to 3 dB greater (*p* < 0.05) than those of SonoVue${}^{\mathrm{TM}}$ and up to 6 dB greater than those of reference bubbles (*p* < 0.001). At 2.25 MHz, antibubble higher harmonics are equivalent to those of SonoVue${}^{\mathrm{TM}}$, with a maximum difference of 1 dB and a *p*‐value above 0.05. Antibubble higher harmonics are up to 7 dB greater than those of reference bubbles, with no overlap of the confidence intervals (*p* < 0.001). At 3.5 MHz, antibubbles scatter higher harmonics that are significantly different from those of reference bubbles and SonoVue${}^{\mathrm{TM}}$: they are up to 2 dB greater than those of reference bubbles (*p* < 0.05) and up to 5 dB weaker than those of SonoVue${}^{\mathrm{TM}}$ (*p* < 0.001).

Comparing plots at MI = 0.2 and MI = 0.3, in several cases, the higher harmonic amplitudes decrease for higher pressures, compared to lower pressures. At 1.0 MHz, we observe this for all contrast agents (*p* < 0.05). For 2.25 MHz, this is observed for SonoVue${}^{\mathrm{TM}}$ and reference bubbles (*p* < 0.05), whereas the signal scattered by antibubbles is equivalent for both sonicating pressures (*p* > 0.05). At 3.5 MHz, SonoVue${}^{\mathrm{TM}}$'s higher harmonics decrease for the higher pressure (*p* < 0.05), whereas those of antibubbles and reference bubbles stay equivalent (*p* > 0.05).

### 3.2 Attenuation

Figure 8 illustrates the mean attenuation of antibubbles, reference bubbles, and SonoVue${}^{\mathrm{TM}}$, with the corresponding standard deviations. For all frequencies, the mean attenuation coefficient is greater for antibubbles, compared to reference bubbles and SonoVue${}^{\mathrm{TM}}$. At 1.0 MHz, antibubbles' attenuation coefficient is slightly higher than that of the reference bubbles, with a mean and significant difference of 1 dB/cm (*p* < 0.05). For other measurements, all the differences in UCA attenuation are significant as well (*p* < 0.001). At 1.0 MHz, the antibubble mean attenuation coefficient is 8.4 dB/cm greater than that of SonoVue${}^{\mathrm{TM}}$. For 2.25 MHz, it is 6.7 dB/cm greater than that of reference bubbles and 9.1 dB/cm than that of SonoVue${}^{\mathrm{TM}}$. At 3.5 MHz, it is 2.6 dB/cm greater that of reference bubbles and 3.4 dB/cm greater than of SonoVue${}^{\mathrm{TM}}$. The antibubbles attenuation coefficients are 8.7, 9.7, and 4.4 dB/cm for 1.0, 2.25, and 3.5 MHz, respectively.

The attenuation measurement conducted in echo mode with the Verasonics system yielded attenuation values of 2.8, 1.1, and 0.4 dB/cm for antibubbles, reference bubbles, and SonoVue${}^{\mathrm{TM}}$, respectively, illustrated with star symbols in Figure 8. The portion of the backscattered pressures $S_{\mathrm{lin}}$ (Equation (4)) constituted 4%, 6%, and 2% of the transmitted pressures for antibubbles, reference bubbles, and SonoVue${}^{\mathrm{TM}}$, respectively.

### 3.3 Dynamic contrast‐enhanced ultrasound

**FIGURE 10:**
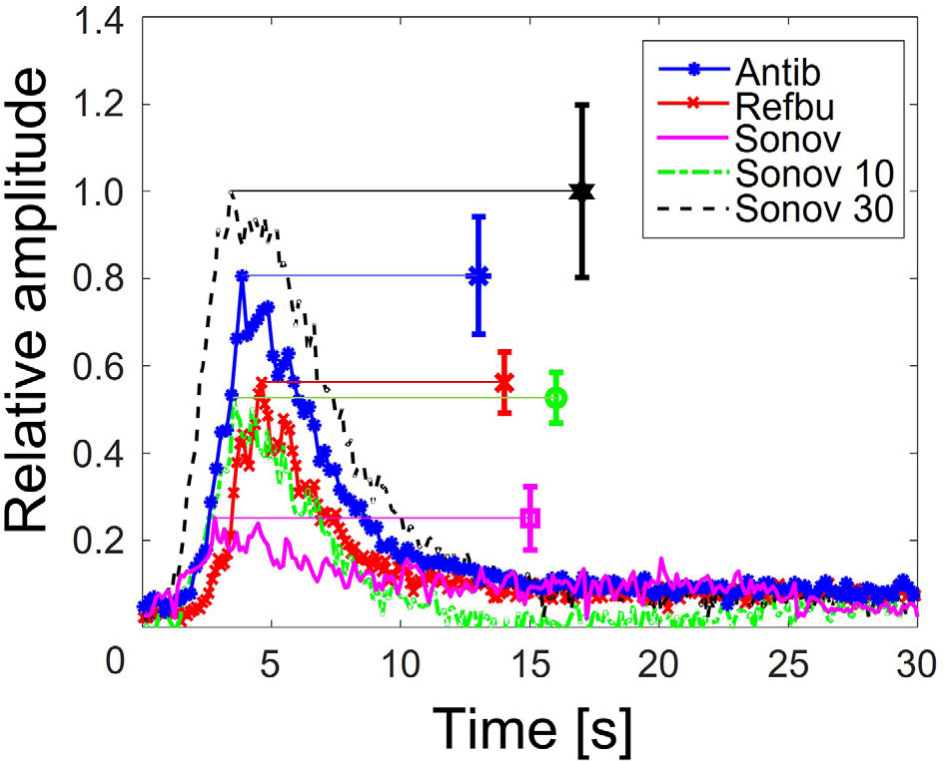
Linearized TICs of the middle region of the vascular phantom, averaged among four acquisitions for all studied dispersions. The peak amplitude for antibubbles, reference bubbles, and SonoVue${}^{\mathrm{TM}}$ is generated by the same concentration of contrast agents as in the static measurement. The peak concentrations of Sonov10 and Sonov30 are 10 and 30 times higher than that in the static measurement. The error bars illustrate the standard deviation at the point of maximum intensity of the averaged curves

Figures 9(a)–(d) demonstrate the maximum intensity projections of the DCE‐US clips recording antibubble, reference bubble, and SonoVue${}^{\mathrm{TM}}$ passage through the employed porous phantom. These images simulate potential clinical images of tissue, when imaging a UCA bolus passage in contrast‐specific mode at pressures inducing nonlinear bubble oscillation. Figure 10 demonstrates the mean linearized TICs of the UCAs, normalized to the maximum mean peak intensity among the UCAs. From the measured TICs, one can observe that antibubbles generate a peak nonlinear signal 31% greater than that of the reference bubbles, 224% greater than that of SonoVue${}^{\mathrm{TM}}$ at the concentration studied in the static measurements, 53% greater than SonoVue${}^{\mathrm{TM}}$ at 10 times the concentration studied in the static measurements, and 23% lower than that of SonoVue${}^{\mathrm{TM}}$ at the highest studied concentration.

## 4 DISCUSSION

The scatter spectra normalized to the saline spectrum (Figure 6) show antibubble higher harmonics to be equivalent or slightly greater, compared to reference bubbles and SonoVue${}^{\mathrm{TM}}$ in the studied frequency range. On the scatter spectra of SonoVue${}^{\mathrm{TM}}$ across all frequencies, we cannot appreciate the expected growth in harmonic amplitude for the frequencies of 2.25 and 3.5 MHz, close to its reported resonance frequency,^30^ in comparison to 1.0 MHz. This underlines that the scatter plots for separate sonicating frequencies do not demonstrate the frequency dependence of the UCA response. The sonicating field is different for every studied frequency, with different dimensions of the focal spot for each source.

For every sonicating frequency, comparing the spectra at MI = 0.2 and MI = 0.3, we could not appreciate any marked growth of the nonlinear components of the scattered signals with increasing pressure amplitude. Markedly, at frequencies of 1.0 and 3.5 MHz, at MI = 0.3, unlike at MI = 0.2, antibubbles scatter a higher nonlinear signal, compared to both other UCAs (*p* < 0.01). The spectrum corresponding to 1.0 MHz and MI = 0.3 exhibits an increase of broadband noise between the second and third harmonic, compared to MI = 0.2, possibly indicating the onset of bubble cavitation.^61^, ^62^ It is also possible that the lower harmonic amplitudes at MI = 0.3 are attributed to the rise of larger bubbles to the surface, because the scatter acquisitions at MI = 0.3 were performed 2–5 s after those at MI = 0.2. Interestingly, antibubbles, the heaviest bubbles, exhibit the lowest difference in harmonic amplitudes for both MIs, whereas SonoVue${}^{\mathrm{TM}}$, the lightest bubbles, exhibit the greatest difference. This indicates that for future characterization, the acquisition at all studied pressures should be performed right after injection of the contrast agents in the cuvette. Alternatively, a thickener can be added to increase the viscosity of the saline, slowing down the rise of larger bubbles to the surface of the dispersion, or a magnetic stirrer could be utilized to keep the dispersion uniform, as in Ref. 48.

The scatter spectra, normalized to the corresponding fundamental signal in the spectrum (Figure 7), show antibubble higher harmonics to be equivalent or up to 3 dB greater than those of reference bubbles. At frequencies of 2.25 and 3.5 MHz, and pressures corresponding to MI = 0.2, SonoVue${}^{\mathrm{TM}}$ scattered the greatest higher harmonic amplitudes among the studied UCAs. This may indicate that, in the given experimental configuration, SonoVue${}^{\mathrm{TM}}$ has a greater capacity to scatter higher harmonics, compared to antibubbles and reference bubbles. At equivalent bubble size and bubble concentration, SonoVue${}^{\mathrm{TM}}$ may generate a stronger nonlinear signal. This might not have been appreciated in Figure 6 due to the smaller size of SonoVue${}^{\mathrm{TM}}$ bubbles or a somewhat lower concentration. On the other hand, the provided normalization cannot fully compensate for the influence of the size distribution, as the energy scattered by certain UCA dispersion is the energy integrated over the bubble size distribution through a complex nonlinear process. Experiments involving other bubble/antibubble size distributions are needed to confirm the observations in Figure 7.

The attenuation measurement is an important indicator of UCA efficacy^28^ because lower attenuation allows avoiding shadowing and imaging at further depth. Endoskeletal antibubbles exhibit the greatest attenuation among the studied UCAs. Given that the attenuation coefficient of most studied dispersions of clinically approved UCAs does not exceed 4.5 dB/cm in the whole diagnostic frequency range,^29^, ^44^, ^63^, ^64^ and that most soft tissue types have an attenuation coefficient below 0.5 dB/(cm MHz),^65^, ^66^ endoskeletal antibubbles exhibit remarkably high attenuation at 1.0 and 2.25 MHz. The high attenuation of antibubbles may be caused by the endoskeleton that may add a viscous behavior to the inner gas phase. Alternatively, the hydrophobic ZnO particles forming the endoskeleton were observed to be surrounded by a thin gaseous layer.^67^ These tiny air pockets are acoustically active at low acoustic amplitudes, absorbing acoustic energy, and cavitating.^67^

The attenuation measurement at 3.5 MHz, conducted with a Verasonics probe in echo‐mode, yielded somewhat lower attenuation values for all UCAs than the through‐transmission measurement with piston transducers. However, both measurements point out the same qualitative differences among UCAs. The discrepancy in the measurement results may be due to different pulse shapes: in the through‐transmission acquisition, a rectangular pulse was utilized, whereas a Gaussian pulse was transmitted in the echo‐mode measurement.

The attenuation measurement is independent of the source pressure field.^30^ The frequency of maximal attenuation of UCAs indicates the resonance frequency of the bubble population.^30^, ^44^ At this frequency, the bubbles transfer a greater portion of energy to higher harmonics. A resonance frequency close to 3.5 MHz is in line with studies that report a resonance frequency close to 3 MHz for SonoVue${}^{\mathrm{TM}}$.^30^ Based on the attenuation measurements, the resonance frequency of reference bubbles is hypothesized to be close to 1.0 MHz, the frequency of maximum attenuation. Following the same reasoning, the resonance frequency of antibubbles is hypothesized to be between 1.0 and 2.25 MHz, closer to 2.25 MHz. This is in line with a smaller size of antibubbles compared to the largest reference bubbles.^29^, ^30^ The presence of an incompressible core also increases the resonance frequency of a bubble.^7^ However, it must be noted that the sonicating pressures differed for the studied frequencies and an additional measurement with equivalent pressures is advisable for future work.

At low acoustic pressures, where mainly linear low‐amplitude bubble oscillation takes place,^7^, ^42^, ^44^ endoskeletal antibubbles backscatter less energy than reference bubbles and more than SonoVue${}^{\mathrm{TM}}$ at the studied concentration, expressed in $S_{\mathrm{lin}}$ (Equation (4)). It is theoretically predicted that the backscattered energy is proportional to the gas volume in the UCA dispersions.^11^, ^29^, ^42^ The same weight of dried contrast material was diluted in saline for antibubbles and reference bubbles, whereas antibubbles contain an endoskeleton and solid cores. This may have resulted in a greater number of reference bubbles than antibubbles, as shown in Figure 2 (81 antibubbles vs. 101 reference bubbles). At the same time, the reference bubble dispersion contains a small percent of reference bubbles almost twice as large as the largest antibubbles (Figure 2). These bubbles have a greater scattering cross‐section.^11^, ^42^ The SonoVue${}^{\mathrm{TM}}$ dispersion clearly contains a smaller gas volume with a comparable number of much smaller bubbles (Figure 1).

The linearized TICs of the DCE‐US acquisition exhibited periodic fluctuations (Figure 10). These can be attributed to the pulsatile flow of the utilized peristaltic pump and to reverberation between the surfaces of the probe and the porous phantom. The TICs showed antibubbles to backscatter a nonlinear signal 31% greater than that of the reference bubbles and 224% greater than that of SonoVue${}^{\mathrm{TM}}$ at the concentration studied in the static measurements. This difference in the scattered signal is not present in the scatter measurement at the corresponding MI of 0.2, where antibubbles and SonoVue${}^{\mathrm{TM}}$ generate equivalent higher harmonics (Figure 6). This finding may indicate that the scatter measurement was masked by the high attenuation of antibubbles surrounding the focal spot. Previous work^23^ and preliminary work support this hypothesis. In preliminary scatter experiments (unpublished data), increasing the concentration of antibubbles and reference bubbles in homogeneous dispersions augmented the scattered nonlinear signal. However, the signal growth with concentration was greater for reference bubbles than for antibubbles. This way, the difference between the amplitudes of the scattered nonlinear signal of reference bubbles and antibubbles decreased with growing concentration. At the same time, in previous work,^23^ a small quantity of antibubbles at a concentration 100 times greater than that in the studied homogeneous dispersions, injected in the very center of the cuvette filled with saline (the location of the peak pressure), generated a second harmonic 10 dB greater than that generated by reference bubbles in the same setting. These findings support the conclusion that, in our scatter measurement configuration, where homogeneous dispersions were used and the US field was focused, attenuation affected the scatter measurement: the advantage of antibubbles over reference and SonoVue${}^{\mathrm{TM}}$ bubbles in Figures 6 and 7 was masked proportionally to the their attenuation. As this evidence is indirect, additional experiments, imaging the fundamental pressure field and the generated second harmonic in the DCE‐US setting, would help clarify whether the effect of attenuation was negligible on the generated and received second harmonic signal. Scatter measurements in a wider/narrower cuvette may identify what role attenuation played in the scatter measurement.

At concentrations 10 and 30 times higher than that utilized in the scatter measurement, the nonlinear response of SonoVue${}^{\mathrm{TM}}$ grows, and at the highest SonoVue${}^{\mathrm{TM}}$ concentration, the antibubble response is 23% lower than that of SonoVue${}^{\mathrm{TM}}$. It is important to note that SonoVue${}^{\mathrm{TM}}$'s resonance frequency is close to 3 MHz, whereas antibubbles have been shown to have a resonance frequency between 1.0 and 2.25 MHz. Therefore, at lower frequencies, when sonicating with plane waves, antibubbles may perform better than SonoVue${}^{\mathrm{TM}}$. Moreover, if made smaller for a clinical application, the antibubble resonance frequency is expected to increase, leading to even greater higher harmonic generation at 3.5 MHz. Figure 9 also illustrates that in cases when small quantities of contrast agent are distributed in a vascular network, in contrast to the scatter measurement, antibubble attenuation of 4.4 dB/cm does not degrade the images in a perceivable manner.

In comparison to commercial UCAs, the studied endoskeleton antibubbles are larger.^1^, ^35^ A size below 7μm in diameter is recommended for UCA bubbles,^35^ about half the size of the largest endoskeleton antibubbles in the studied suspensions (Figure 2). The shell thickness of commercial UCAs ranges from 2 to 200 nm,^1^, ^16^, ^25^, ^68^ with SonoVue${}^{\mathrm{TM}}$ having a particularly thin and compliant shell of about 4 nm.^25^ The silica shell of antibubbles is stiff and about 1μm thick, based on bright field microscopy images of antibubbles with the same shell.^7^ The shell properties greatly contribute to UCA attenuation,^11^, ^43^ suggesting a study of alternative compliant materials for the antibubble shell that would reduce shadowing effects associated with high antibubble attenuation. Current generation contrast agents such as SonoVue${}^{\mathrm{TM}}$ and Definity${}^{\mathrm{TM}}$ typically contain low‐solubility gas, providing a longer bubble lifetime. The studied endoskeleton bubbles contain highly soluble air. No endoskeleton or core structures are present in any of the currently approved contrast agents.

## 5 CONCLUSIONS

Based on the previous work, antibubbles, that is, encapsulated gas bubbles with incompressible cores, are expected to demonstrate augmented nonlinear behavior compared to encapsulated gas bubbles. This opens the door to improving CE‐US image quality and to a traceable therapeutic agent with large amounts of therapeutic compounds in the core. This work aimed at characterizing the nonlinear behavior of endoskeletal antibubbles, an antibubble UCA prototype, and comparing it to reference bubbles and a commercially available and clinically approved UCA, SonoVue${}^{\mathrm{TM}}$, in the range of diagnostic frequencies from 1.0 to 3.5 MHz and pressures comparable to those employed clinically at MI = 0.2 and MI = 0.3.

We demonstrated that the studied dispersions of endoskeletal antibubbles generate comparable or greater higher harmonic content than those composed of reference bubbles with an equivalent median diameter and smaller SonoVue${}^{\mathrm{TM}}$ bubbles. Higher harmonics comparable to that of SonoVue${}^{\mathrm{TM}}$ at a high concentration may be attributed to a larger antibubble size, a different shell, and gas. Figure 7 mitigates the influence of bubble concentration and bubble size on the scattered spectra. However, the signals scattered by the UCAs in the focal spot may have been attenuated by the surrounding UCA. Therefore, the advantage of antibubbles over other contrast agents may have been masked in Figure 7 by their higher attenuation. The plane‐wave DCE‐US measurement, simulating clinical imaging at 3.5 MHz, demonstrated that antibubbles have comparable performance to SonoVue${}^{\mathrm{TM}}$ at a high concentration at a frequency close to its resonance frequency, and superior performance, compared to the reference bubbles.

Based on this work, it is difficult to draw the solid conclusion that the incompressible core leads to greater higher harmonic generation, due to the differences in the size distributions (Figure 2) and inner bubble content of the studied antibubbles and reference bubbles (Figure 1). The stronger higher harmonics of antibubbles, compared to reference bubbles, may also be attributed to the lower resonance frequency of the reference bubble dispersion. To present proof that antibubbles have an advantage over bubble‐based contrast agents for imaging purposes, other reference bubbles are required, identical to antibubbles in all aspects, except for the core. Such an agent is currently not available. Nevertheless, we present evidence that endoskeletal antibubbles demonstrate strong nonlinear behavior at frequencies from 1.0 to 3.5 MHz. These results are encouraging and suggest that antibubbles hold high potential to serve as traceable therapeutic agents. For this purpose, the solid core would have to be replaced by a liquid inclusion with medication.

## CONFLICT OF INTEREST

The authors have no conflicts to disclose.

## Data Availability

The data that support the findings of this study are openly available in Zenodo at https://doi.org/10.5281/zenodo.5514221.
